# Observations on the Efficacy of the White Mustard Seed in Affections of the Liver, Internal Organs, and Nervous System

**Published:** 1828-04

**Authors:** 


ART. III.—*Observations on the efficacy of White Mustard-seed, in affections of the Liver, Internal Organs and Nervous System; and on the general management of health and life. By* CHARLES TURNER COOKE, *Member of the Royal College of Surgeons. First American, from the fourth English Edition. New-York*, 1827, 18*mo. pp.* 116.

The plants of the natural order Cruciferæ are remarkable for containing a pungent, acrid and volatile principle. In most of the species it is found in the seeds; and this is the case, in the different kinds of mustard, of which two, sinapis nigra and sinapis alba, the black and white, have, from time immemorial, claimed to be admitted into the apothecary's shop, not less than the larder of the gourmand. In support of their pretensions, to *officinal* distinction, many eulogistic paragraphs have at different times been written; but, except for external application, or in "patent essences," they have, on the whole, not attracted a degree of attention corresponding to the praises which have been lavished upon them.

In 1693, the testimony of Dr. Salmon was as followeth:—Referring to the seed, "It is," says he, "hot and dry, incides, attenuates, attracts, rubefies, is stomachick, pectoral, and nephretic, provokes appetite, is good against hypochondriac melancholy, quotidians, and quartans, from a tartareous mucilage cures dropsies, and is good against the stone." But these are only a part of his virtues, and he refers to the pharm. Lond. lib. I. cap. 7, sect. 120, for the remainder; concluding his article with the remark, that a "sinapism put into the nostrils drives away the lethargy."

Great, however, as were the virtues of mustard seed, when administered to the people of England in 1693, they were nothing in comparison with what Dr. Turnor has found them, as late as 1823. His publication, which constitutes the first article of the work before us, presents the following statement:

"The White Mustard Seed is an almost certain remedy for all diseases connected with disordered functions of the stomach, liver and bowels, and *as such* has been eminently successful in the following (among other) cases, viz.—in tendency of blood to the head, headache, weakness of the eyes and voice, and hoarseness; in asthma, shortness of breath, wheezing, cough, and other distressing affections of the chest; in indigestion, oppression after eating, heartburn, sickness, wind and spasms, cramp and other uneasy affections of the stomach; in debility, uneasiness, pain and sense of tenderness and soreness in the interior, and particularly at the pit of the stomach, and in pain in the sides and lower part of the body; in scanty and redundant flow of bile, in obstructions that may lead to scirrhous liver, torpor and other morbid affections of that organ; in deficient perspiration, gravel, scanty and unhealthy state of the urine, and other disorders of the skin and kidneys; in relaxed and irritable bowels, flatulence, and occasional and habitual costiveness; in severe colds, rheumatism, lumbago, spasms and cramp in the body and limbs, partial and general dropsy, palsy, coldness and numbness of the limbs and feet, loss of appetite, failure of sleep, weakness of nerves, depression of spirits, and general debility of the system. In ague, gout, rheumatic fever, epilepsy, scrofula, scurvy, erysipelas or St. Anthony's fire, in the dreadfully painful affection called tic douloureux, and in recovery from the small pox, typhus and scarlet fevers, and other severe disorders connected with a depraved state of the interior—it has been taken with very considerable advantage. For the long round worms and the small white ones also, it is incomparably the best remedy hitherto discovered; inasmuch as both in children and grown-up persons, it not only destroys those reptiles, but if persevered in long enough to restore the tone of the stomach and bowels, will prevent their recurrence in future." pp. 10—11.

The author of the Theriacā Andromachi, arrayed sixty articles combined, against the bite of the viper; Mr. Turnor, on the other hand, opposes one remedy to more than sixty maladies. That this is a formidable group to be scattered by mustard seed, cannot be denied. Mr. Cooke himself seems to have thought so; and a leading object of his work is to explain the manner in which this end is accomplished. Forty pages of his little book are accordingly devoted to the remote causes and nature of the disorders in Mr. Turnor's Nosology. The former are vicissitudes in climate, excessive drinking, gluttony, and the 'play of the passions;' the effects of which are, chiefly, great excitement in the digestive organs, followed by torpor, deranged sensibilities and depraved secretions, especially in the liver; whence are propagated, by sympathy, to various parts of the body, the morbid changes which eventuate in dropsy, rheumatism, apoplexy, and the other maladies of the catalogue.

It is to remove this condition of the digestive system, the occasion of so many woes, that Messrs Turnor and Cooke, administer the White Mustard. According to Mr. C. three primary indications are to be followed:

> " 1st.—To increase and meliorate the biliary fluid.
> 2d.—To procure the daily removal of the vitiated secretions of the liver and other digestive organs.
> 3d.—To increase the tone and digestive power of the alimentary canal." pp. 62—63.

And to the fulfilment of these, he considers the White Mustard to be exactly adapted.

> "It is," says he, "at once a *Tonic*, in the best sense of the term—an *Aperient* of unrivalled superiority—and a *Sedative*, of the most soothing and salutary kind. And in this way it is that it produces its three-fold kindly office: 1st, by yielding a considerable quantity of a mild assimilating mucilage most friendly to an irritable state of stomach and bowels: 2dly, by its gradually and gratefully stimulating effects upon the whole interior surface of both: and 3dly, by its slightly mechanical action, assisting in the propulsion of their contents. Hence it at the same time strengthens and invigorates in a very remarkable degree the *whole line* of the alimentary canal, and consequently improves the digestion and assimilation of food; and with these the appetite, sleep, and general health. To the poor it is invaluable in every point of view; to them it is both food and medicine, and is on this account peculiarly calculated to meet the numerous and formidable physical evils with which they have to contend, and to which they are peculiarly exposed. It is a remedy adapted at once to infancy and to old age. It enables the young to struggle against the morbid debility frequently attaching to their tender years, and it supports the aged under the pressure of those infirmities generally annexed to declining life—whilst in every stage of existence, and in every scale of being, it would seem to impart the power of resisting the effects of sudden changes of atmosphere, and thus to obviate that host of evils, which flow from our variable and uncertain climate." pp. 72—75.

Speaking of himself, he says—

"I can declare with truth, that I never knew what it was to enjoy a feeling of comfortable health unbroken for twenty-four hours together, until I became acquainted with the "efficacy of," and "took whole," "White Mustard Seed." p. 78.

In ordinary causes, the seed, unbroken, is to be given three times a day in tea, or dessert spoonful doses. Even a table spoonful may be required. It must be made to operate gently on the bowels; and when it fails in this effect, a dilute solution of Epsom salt, or some other laxative, must be added. The diet of the patient should be temperate and regular, and his skin, well defended from cold and moisture, subjected to frictions and baths. In certain cases a gentle mercurial course must be previously employed.

We shall conclude with a few admonitory remarks.

As many of the disorders for which the white mustard is recommended, are the offspring of excessive stimulation, of one kind or another, it is not likely that they can be cured, indiscriminately, by an acrid stimulant. They are, in part at least, the offspring of the excessive use of *black* mustard and other condiments; and Dr. C. proposes to cure them by the use of *white* mustard, which differs from the other, only, in being a little milder! If, in reality, it possesses the active powers ascribed to it by our athor, it must be either useless or prejudicial in a great number of the diseases for which he recommends it, when they occur in early or middle life, and in persons of a sanguine temperament. To those of a phlegmatic temperament, with slugglish fibres, and torpid bowels; to habitual drunkards, if actual inflammation has not been awakened in any organ; to dyspeptics, who have been long afflicted; to the aged; and, finally, to those with broken-down constitutions, from almost any cause—the white mustard, or even the black, may be given, not only with impunity, but with a good prospect of success; and to all such, we beg leave to recommend it, not quite in the language of Mr. C.—"as one of the most decided discoveries of general usefulness and applicability, which has ever been made known—one of the greatest blessings that has ever been dispensed to suffering man;" which, it may be expected, "will considerably lengthen human life in this kingdom, and, finally, be adopted throughout the world"—but as a cheap and convenient laxative, of the stimulating class.

